# A Case of Prefemoral Fat Pad Impingement Syndrome Caused by Hyperplastic Fat Pad

**DOI:** 10.1155/2018/3583049

**Published:** 2018-12-23

**Authors:** Suguru Koyama, Keiji Tensho, Hiroki Shimodaira, Tomoya Iwaasa, Hiroshi Horiuchi, Hiroyuki Kato, Naoto Saito

**Affiliations:** Department of Orthopedic Surgery, Shinshu University School of Medicine, 3-26-1, Asahi, Matsumoto, Nagano 390-8621, Japan

## Abstract

**Case:**

We report a rare case of prefemoral fat pad impingement syndrome that was caused by a hyperplasia of the normal suprapatellar fat pad. Pain and catching were observed in the proximal-lateral patellofemoral joint, and MRI imaging confirmed a hyperplasic mass in the same area. Although conservative treatment showed no signs of improvement, symptoms improved after an arthroscopic excision of the mass.

**Conclusion:**

Prefemoral fat pad impingement syndrome is related to patellar motion and should be considered as one of the underlying causes of anterior knee pain (AKP). Surgeons should recognize that a small hyperplasia composed of normal adipose tissue can cause AKP.

## 1. Introduction

Prefemoral fat pad impingement syndrome (PFIS) is considered as one of the underlying causes of anterior knee pain (AKP) [1–5]. PFIS is generally induced by tumorous lesions that occur at the same site, such as lipomas and lipoma arborescens, causing the fatty tissues of the anterodistal femur to impinge. However, there are very few reports on PFIS induced by normal adipose tissue. We report a case of PFIS caused by normal adipose tissue, located superolaterally to the patellofemoral (PF) joint and within the suprapatellar pouch, which was excised under arthroscopic examination.

## 2. Case Presentation

A 49-year-old male patient was presented at our hospital with a difficulty in ambulation. Three days prior, the patient experienced discomfort in the anterior knee joint with no preceding injury, and the symptom progressed into pain the following day. There was no swelling of the joint, but the patient showed severe restriction in the range of motion due to pain. There was no tenderness at the medial and lateral femorotibial (FT) joint, and tenderness was only observed on the proximal side of the PF joint. The Lachman test, pivot-shift test, varus/valgus instability, and McMurray test were negative. Although symptoms temporarily improved with an intra-articular injection of xylocaine, catching of the proximal-lateral knee was subsequently observed while moving the leg from full extension to flexion.

**Figure 1:**
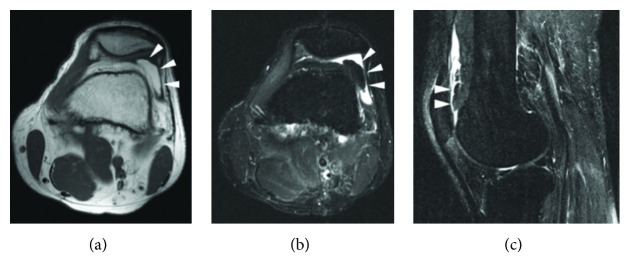
Preoperative MRI. Axial T1 MRI (a), axial STIR MRI (b), and sagittal STIR MRI (c) revealed the prefemoral fat pad (white arrowhead). The fat pad was located superolaterally to the PF joint, and hyperplasia was confirmed. There were no fibrous septa or mild peripheral enhancement patterns that were suggestive of lipoma.

Simple radiographs showed no abnormal findings, and MRI images revealed a soft tissue mass located superolaterally to the PF joint that exhibited an ill-defined border with its surroundings. Both T1- and T2-weighted images at high signal intensities revealed the soft tissue mass, while low signal intensity was noted under fat suppression and no contrast enhancement was noted under contrast imaging (Figure 1). In addition, there were no abnormal findings in the blood examination.

**Figure 2:**
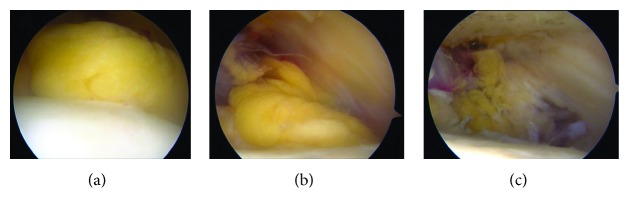
Intraoperative arthroscopic view. (a, b) Arthroscopic view of the anterolateral portal before excision of the prefemoral fat pad. The fat pad resembled normal adipose tissue, and there were no characteristics of lipoma such as round/oval masses, fibrous capsules, or vascular pedicles. Mild laxity was observed. (c) A piece-by-piece excision of the prefemoral fat pad was performed.

**Figure 3:**
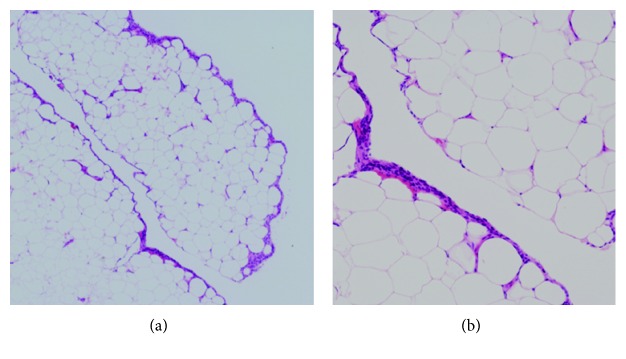
Histological findings consisted of connective tissues that were mainly composed of fat, and synovial covering was not inflamed. There were no fibrous capsules, vascular pedicles, or villous projections that were suggestive of lipoma, lipoma arborescens, or pigmented villonodular synovitis (hematoxylin and eosin (HE); magnification: (a) ×40, (b) ×100).

The patient requested surgery due to his persistent symptoms and underwent knee arthroscopy. A large fat mass with mobility in the proximal to distal direction was observed on the anterior surface of the proximal-lateral PF joint. The mass macroscopically resembled fat, and the border with its surroundings was ill-defined. There were no other intra-articular findings, and a piece-by-piece resection was eventually performed (Figure 2). Histologically, the mass consisted of connective tissue that was mainly composed of fatty tissue, and there were no findings that suggested the presence of lipoma, lipoma arborescens, or pigmented villonodular synovitis (Figure 3).

**Figure 4:**
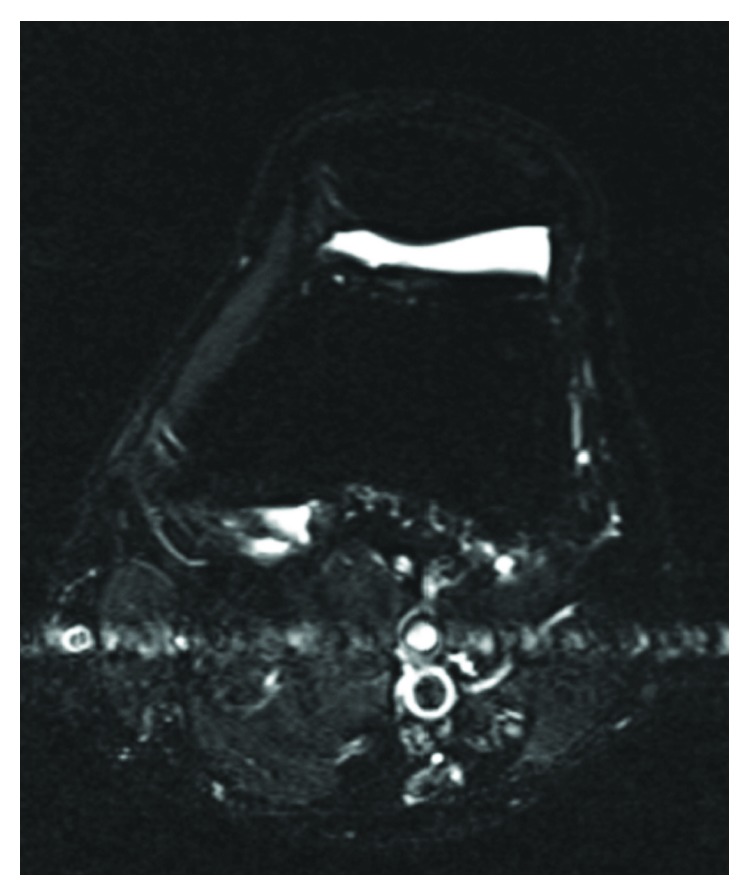
10-month postoperative axial STIR MRI. The fat pad was completely removed and no recurrences were found.

The symptoms improved after surgery, and no symptoms were found thereafter, including anterior knee pain (AKP) at 3 years after surgery. There was no evidence of the preoperatively confirmed fatty mass in postoperative MRI imaging, and no recurring lesions were found (Figure 4).

## 3. Discussion

Along with the quadriceps (anterior suprapatellar) and infrapatellar (Hoffa's fat pad) fat pads, the prefemoral (posterior suprapatellar) fat pad is one of the three major anterior knee fat pads, and the structure consists of fat cells that are found in the suprapatellar bursa, located superior to the femoral trochlea and the anterior cortex of the distal femoral metaphysis [1–4]. These fat pads are composed of deformable fat/fibrous tissues and are responsible for synovial fluid production while acting as a protective material for the joint surface [1, 4]. However, recurrent chronic impingement, trauma, and instability of the PF joint are known to cause degeneration and hyperplasia of the fat pad that may lead to AKP, among which is the PFIS, an impingement caused by the anterior surface of the femur, adipose tissue, or a tumor [1, 4–6]. Compared to the quadriceps and infrapatella fat pads, there are few reports of PFIS which are predominantly caused by lipoma or lipoma arborescens. To the best of our knowledge, only one case of PFIS caused by normal adipose tissue has been reported by Kim et al. [5].

**Figure 5:**
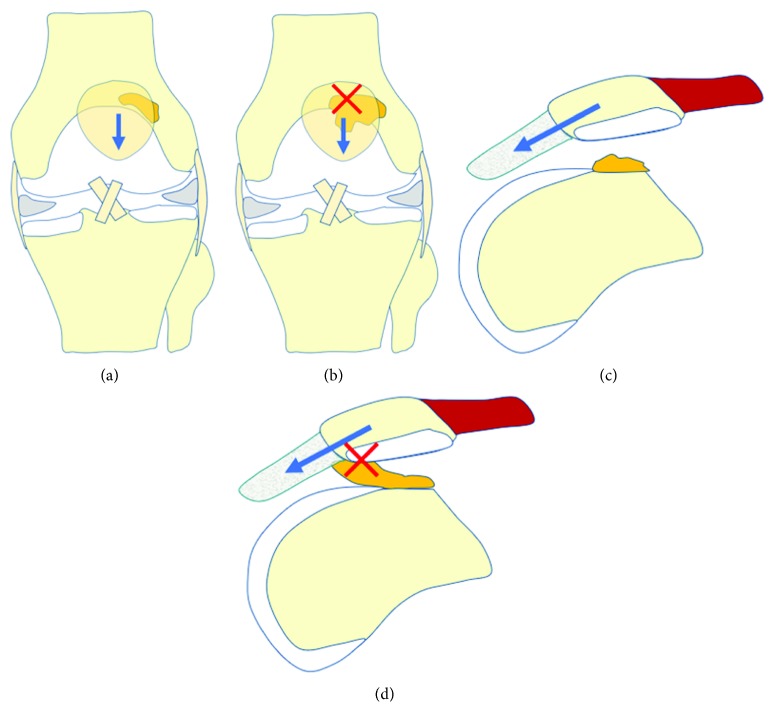
Anatomical movement of the patella from full extension to flexion. (a) In the anterior view, the patella moves from the proximal to the distal center and engages with the trochlear groove. (b) When the fat pad is large, PFIS occurs on the proximal side of the patella. (c) In the lateral view, patella moves distally as the knee flexes. A normal-sized prefemoral fat pad does not demonstrate impingement. (d) When the prefemoral fat pad is larger than normal with good mobility, the fat pad can get caught by the PF joint regardless of whether the patella shows normal movement, resulting in symptoms.

In this case, patellar movement is thought to be related to the onset of PFIS. Anatomically, when the knee is fully extended, the patella is located on the proximal position of the femoral trochlea and does not come into contact with the trochlear groove. As the knee is flexed, the patella moves towards the center of the PF joint to engage and stabilize within the trochlear groove (Figures 5(a) and 5(b)). Because the contact surface of the patella side moves from the distal to proximal direction and that of the trochlea side moves from the proximal to distal direction as the knee is flexed, these movements act as though to “sandwich” the prefemoral fat pad on the anterolateral surface of the femoral cortex. Although this movement was not arthroscopically confirmed, when the prefemoral fat pad is larger than normal, regardless of whether the movement of the patella itself is normal, the fat pad can get caught by the PF joint as the knee is moved from full extension to flexion, possibly resulting in pain (Figures 5(c) and 5(d)). In our present case, the normal adipose tissue on the anterolateral surface of the femur was unusually large, and we observed a mild laxity due to the adipose tissue under arthroscopy. Although the cause of the acute onset was unclear, we suspect that the following overlapping conditions had led to changes in the mobility of the fat pad, resulting in the recurrent catching of the knee: (1) the prefemoral fat pad was natively larger than in normal patients and (2) the fat pad was positioned where it is easily caught by the PF joint and was caught for the first time. Similar forms of mechanical impingements have also been reported in cases of lipoma arborescens [7]. Although there is a report that the patella alta is associated with prefemoral fat pad synovitis [8], the Insall-Salvati ratio was 1.0 in this case, and we did not find morphological abnormalities of the patellofemoral joint, including the patella alta.

PFIS includes various symptoms such as AKP, synovitis, restricted range of motion, joint swelling, and pain/catching of the proximal patella due to the movement of the knee joint [1, 4, 5]. In terms of image findings, MRI is useful and can serve to confirm tumorous or abnormally thick adipose tissue on the proximal-anterolateral femur of the PF joint [1–5]. However, the presentation of signal change is not uniform. Although the mass presents a high signal on fat-suppressed images in cases with inflammation or bleeding [1, 4], as demonstrated in this case and a report by Kim et al. [5], attention should be paid to the fact that some cases show the same signal as adipose tissue. In addition, there is a report that suggests that there is little relevance between AKP and signal changes accompanied by edema [9]. Diagnosis is difficult based on symptoms or imaging alone, and definitive diagnosis is ultimately confirmed when symptoms are improved as a result of resecting the lesion. Conservative treatment did not show improvement in this case, and the disease was likewise confirmed by symptomatic improvement by resecting the lesion.

The differential diagnosis includes intra-articular lipoma (IL), lipoma arborescens (LA), and localized pigmented villonodular synovitis (PVNS) [1, 5, 7, 10–14]. If mild hyperplasia is observed, surgeons should be aware of pathological conditions in which the fat pad may impinge and cause pain on the PF joint during full extension to flexion of the knee, even if the fat pad consists of normal adipose tissue. In light of this present case, we believe that it is necessary to perform a comprehensive differential diagnosis of PFIS with clinical, imaging, arthroscopic, and histological findings when identifying the cause of AKP.

We reported a case of PFIS of the fat pad that presented with a hyperplasia of normal adipose tissue. Although an accurate diagnosis was challenging to obtain, a definitive diagnosis was achieved when symptomatic improvements were observed after resection. Surgeons should recognize that subtle morphological abnormalities may cause pain at the same site even in normal tissues.
